# Identifying the Phenotypic and Temporal Heterogeneity of Knee Osteoarthritis: Data From the Osteoarthritis Initiative

**DOI:** 10.3389/fpubh.2021.726140

**Published:** 2021-08-19

**Authors:** Mengjiao Li, Lan Lan, Jiawei Luo, Li Peng, Xiaolong Li, Xiaobo Zhou

**Affiliations:** ^1^West China Biomedical Big Data Center, West China Hospital/West China School of Medicine, Sichuan University, Chengdu, China; ^2^Surgery, Civil Aviation Medical Center Chengdu, Chengdu, China; ^3^School of Biomedical Informatics, The University of Texas Health Science Center at Houston, Houston, TX, United States

**Keywords:** osteoarthritis, phenotype, subtype, progression, trajectories

## Abstract

**Objective:** Previous studies discussing phenotypic and temporal heterogeneity of knee osteoarthritis (KOA) separately have fatal limitations that either clustering patients with similar severity or assuming all knees have a single common progression pattern, which are unreliable. This study tried to uncover more reliable information on phenotypic and temporal heterogeneity of KOA.

**Design:** Data were from Osteoarthritis Initiative database. Six hundred and seventy-eight unilateral knees that have greater Kellgren and Lawrence (KL) grade than the contralateral knees at baseline and in all follow-up 48 months were included. Measurements of biomarkers at baseline were chosen. Subtype and Stage Inference model (SuStaIn) was applied as a subtype-progression model to identify subtypes, subtype biomarker progress sequences and stages of KOA.

**Results:** This study identified three subtypes which account for 15, 61, and 24% of knees, respectively. Each subtype has distinct subtype biomarker progress sequence. For knees with KL grade 0/1, 2, 3, and 4, they have different distributions on stage and 26, 53, 89, and 95% of them are strongly assigned to subtypes. When assessing whether a knee has KL (grade ≥ 2), subtypes and stages from subtypes-progression model (SuStaIn) are significantly better fitting than those from subtypes-only (mixture of Gaussians) (likelihood ratio = 105.59, *p* = 2.2 × 10^−16^) or stages-only (SuStaIn where setting *c* = 1) (likelihood ratio = 58.04, *p* = 2.57 × 10^−14^) model. Stages in subtypes-progression model has greater β than stages-only model. Subtypes from subtypes-progression model have no statistical significance.

**Conclusions:** For subtypes-progression model, stages contain more complete temporal information and subtypes are closer to real OA subtypes.

## Introduction

Knee osteoarthritis (KOA) is recognized as a complex condition with different clinical characteristics (1–3). Most of previous studies only discussed phenotypic or temporal heterogeneity of KOA, which referred as a subtypes-only models or stages-only models.

Subtypes-only models cluster knees together into subtypes based on the similarity of the biomarker measurements (1, 4). The limitation is that those models could result in clusters of patients with similar osteoarthritis (OA) severity, which would not represent true OA subtypes. Stages-only models are always built based on regression model (5–8). The inherent assumption is that all knees have a same single common progression pattern. But disease progressions in most cases are complex and knees have phenotypic heterogeneity. Therefore, stages identified based on the above assumption have limited reliability. Some researchers tried to investigate subtypes and temporal heterogeneity together (2, 9). However, they discussed the distinct subtypes and OA severity scores separately. The above-mentioned limitations could not be avoided in previous studies.

KOA is a chronic progressive disease and has a long course. The ideally long-term frequent follow-up data are difficult to obtain. In this study, we use cross-sectional data to research the characteristics of KOA progression and phenotypic heterogeneity. There are three basic assumptions for this study. (1) Cross-sectional data contain a certain amount of temporal information. Knees have different changes in biomarker measurement, which implies the disease stage that they belong to. (2) Cross-sectional data consists of subjects at all the periods of the whole disease course. It can be roughly affirmed by the knees distributed in all the KL grades. The first two assumptions make it possible that researchers can reconstruct the trajectory of disease progression with cross-sectional data. (3) Knees from different subtypes have different trajectories of biomarker progression. Thus, the optimal number of biomarkers progression trajectories that maximizes the data likelihood represents the optimal number of subtypes.

New machine learning and deep learning methods (10–12) are brought into medical research. The recently introduced Subtype and Stage Inference model (SuStaIn) is an unsupervised machine-learning technique (13) and learns distributions of biomarker values from the data. SuStaIn calculates the optimal subtype biomarker progress sequences and the optimal number of sequences to maximize the data likelihood. The number of subtypes is represented by the optimal number of sequences. The subtype biomarker progress sequence stands for the order of the biomarker changed as disease progresses for a particular group of knees. With the subtype biomarker progress sequences, knees can be assigned into a certain subtype and progression stage. Therefore, we used SuStaIn as the subtypes-progression model to uncover phenotypic and temporal heterogeneity of KOA simultaneously. And finally, we identified 3 KOA subtypes, rebuilt the subtype biomarker progress sequences and assign each individual to a most probable subtype and stage.

## Materials and Methods

### Data Description

#### Study Population

The data were from Osteoarthritis Initiative (OAI) database (https://nda.nih.gov/oai). OAI is a large multi-center, 10-year prospective observational cohort study. The original OAI participant recruitment and data collection process have obtained ethical approval and informed consent. No specific ethical approval was required for this study.

Previous study shows that risk of KOA increased with the incidence of contralateral knee OA (3). For the two knees of each subject which afflicted with KOA earlier and later, there may be different risk factors and disease progression patterns. As limited by available number of knees, we decided to discuss a single condition that the unilateral knees have greater Kellgren and Lawrence (KL) grade than the contralateral knees at baseline and in all the follow-up 48 months. The exclusion criteria are knees (1) having no KL grade assessment at baseline or in any follow-up visit, (2) having no complete data of radiograph and MRI image assessment at baseline. Finally, our study population includes 678 eligible knees with different KOA severity.

Obviously, to construct the subtype biomarker progress sequences, the data should cover the whole disease course of KOA. Ideally, it should contain complete biomarkers' measurements in the whole disease course of patients. However, the course of KOA, just like other chronic diseases, takes a period of decades. Following up for the whole disease course is impossible. Study the disease trajectories with only cross-sectional data is necessary. Then, we only chose the measurements of biomarkers at baseline for each knee. Because knees' KOA severity increases over time and the number of knees with mild severity reduces at follow-up time points. KOA severity of study group ranges more widely at baseline than any follow-up time points.

#### Biomarkers

Candidate biomarkers used in this study were the OA symptoms which were obtained through questionnaires [Western Ontario and McMaster Universities (WOMAC) pain score], quantitative radiographic readings [Joint Space Width (JSW)], quantitative MRI measures of cartilage thickness, and semi-quantitative radiographic readings (osteophytes and sclerosis, per anatomical compartment for the tibia and femur).

To simplify the model, increase the clinical utility and improve the generalization ability the most, we used the backward deletion to select the fewest biomarkers, which maximized the data likelihood (14). All the biomarkers were used to reconstruct KOA subtypes progression models shown in Table 1 and **Figure 2A**.

**Table 1:** Detailing the biomarkers used in the study.

**No**.	**Biomarkers**	**Number of z-scores events**	**Maximum z-score**
1	**WOMAC pain score**	3	5
2	**Medial minimum JSW**	3	5
**Osteophytes**			
3	Tibia medial subregion	3	3
4	Tibia lateral subregion	2	3
5	Femur lateral subregion	3	3
6	Femur medial subregion	2	3
**Sclerosis**			
7	Femur medial subregion	2	3
8	Tibia medial subregion	1	3
**Mean cartilage thickness**			
9	Central medial femur condyle (external)	3	5
10	Central medial femur condyle (center)	3	5
11	Central medial femur condyle (internal)	2	2
12	Central lateral femur condyle (internal)	2	3
13	Central lateral femur condyle (external)	3	3

*The order numbers of biomarker in Table 1 match the numbers in Figure 2A*.

### Study Roadmap

**Figure 1:**
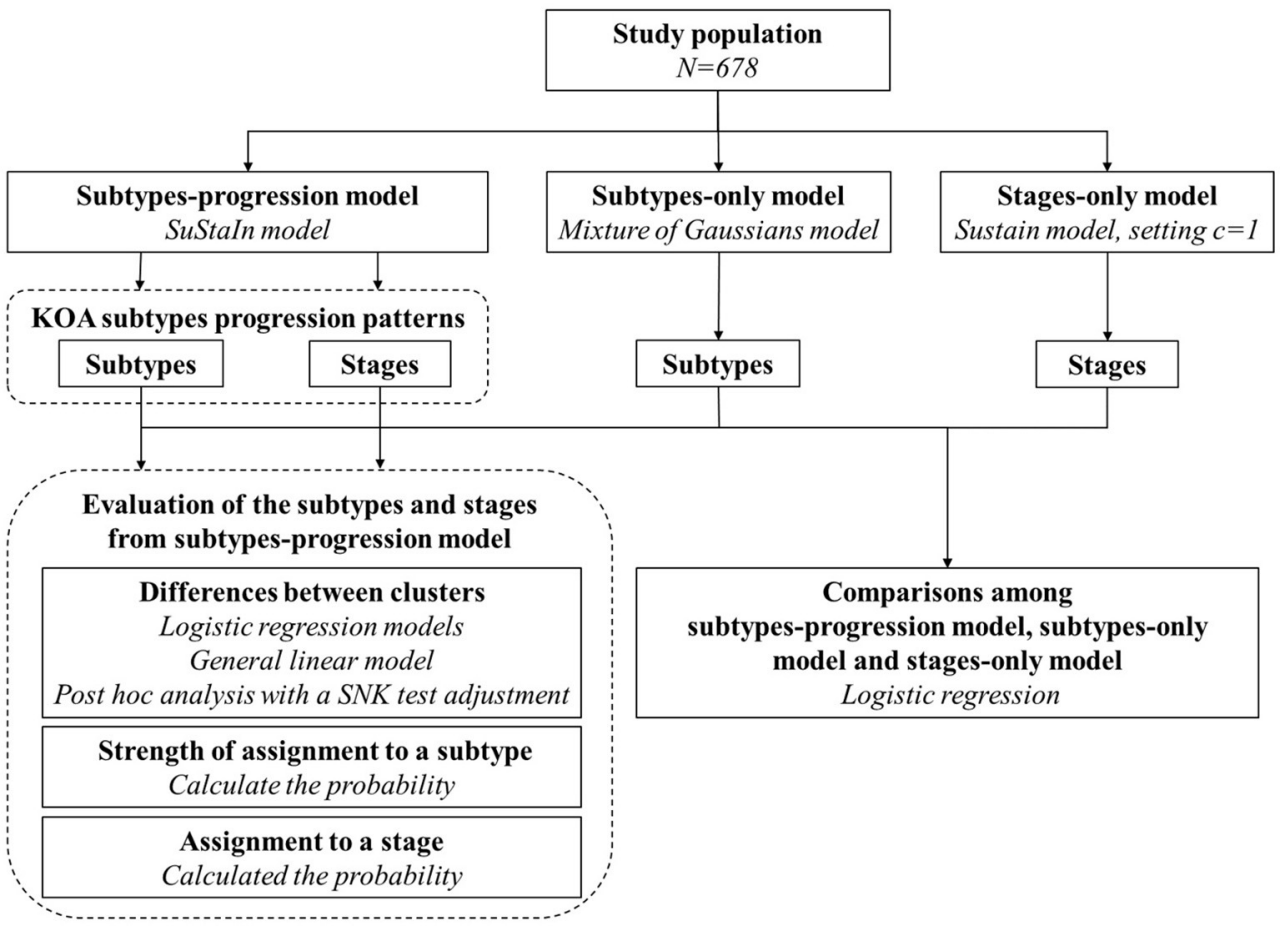
Study roadmap for our study.

The study roadmap is shown in Figure 1. We included 678 knees in the study population. Measurements of biomarkers at baseline were chosen. Three models (SuStaIn model, mixture of Gaussians model and SuStaIn model where setting *c* = 1) were fitted to the study population and formulated the subtypes-progression model, subtypes-only model, and stages-only model, respectively. The subtypes, subtype biomarker progress sequences and stages from the subtypes-progression model were described and assessed. Finally, we compared the subtypes and stages among the three models.

### Subtypes-Progression Model

#### KOA Subtypes, Subtype Biomarker Progress Sequences, Stages, and SuStaIn Model

In this study, we used SuStaIn as the subtypes-progression model to identify KOA subtypes, rebuilt the subtype biomarker progress sequences and assigned an individual to a most possible subtype and stage. SuStaIn defined four important concepts. (1) Biomarker event. A biomarker event is a new change in symptom or structural lesion as KOA progression advances. Each biomarker event corresponds to a switching from a z-score to another. (2) Subtype. knees from different subtypes have different trajectories of biomarker progression. The number of biomarkers progression trajectories can be discovered in a disease represents the number of all subtypes it contains. (3) Subtype biomarker progress sequence. Each disease subtype has a particular progress course. The course of each disease subtype progression can be depicted by the order in which the biomarker events occur as each disease subtype progresses, which is called as subtype biomarker progress sequence. (4) Stage. Each disease subtype progression advances from 0-th stage to S-th stage. S is the number of all biomarker events. The i-th stage that a knee belongs to is defined as a specific state that the previous i events of the sequence have occurred. Occurrence of an event indicates that the disease advances in a biomarker and disease progression switches from a stage to the next.

SuStaIn is an unsupervised machine-learning technique and doesn't rely on a priori staging or subtype. SuStaIn is a mixture of linear z-score models. It describes the subtype biomarker progress sequence as a linear z-score model, which is an improved model of the original event-based model (EBM) (15, 16). In EBM, each event represents the switch of a biomarker from a normal to an abnormal level. SuStaIn reformulates the events to make them correspond to the continuous linear accumulation and each event of a biomarker represents change from one z-score to another. SuStaIn simultaneously optimizes the number of subtypes, subtype biomarker progress sequence, and the posterior distributions of both. What's more, SuStaIn estimates the probability of assignment to a most probable subtype and stage, respectively, for each knee. The most likely biomarker event sequences are ones that maximizes the data likelihood. The optimal number of subtype biomarker progress sequences that maximize the data likelihood represent the optimal number of subtypes. We fitted SuStaIn with python (version 3.7). The source code of SuStaIn can be acquired on https://github.com/EuroPOND/pySuStaIn.

#### Data Pre-processing

Every biomarker measurement was expressed as a z-score relative to the control group. Since this, the corresponding z-score can describe the abnormal degree of each biomarker measurement from study population relative to control group. Inclusion criterion for the OAI control group are (1) no pain, aching or stiffness in both knee in the past year; (2) no radiograph OA; (3) no eligibility risk factors; (4) age ≤ 70 years. This study had an additional exclusion criterion: incomplete data on radiographic and MRI image assessment at baseline.

With KOA progress, medial minimum JSW and mean cartilage thickness decrease and their z-scores became negative. We took the negative value of the z-scores for convenience, so that all the z-scores would increase as KOA severity increasing.

#### Input

A biomarker event is a new change in symptom or structural lesion as KOA progression advances. Each biomarker event corresponds to a switching from a z-score to another. The z-score events of each biomarker were the most important input. For each biomarker, the z-score events initially include z-scores of 1, 2, 3, and 5. However, some z-score events had low occurrence frequency in the disease progression, thus fewer than 10 knees had greater than that z-score. To simplify the model, we excluded those z-score events. Finally, 32 z-score events were included from the 13 biomarkers.

The maximum z-score means the final stage of the progression. If the maximum z-score event was 1, 2, 3, and 5, the input “maximum z-score” was set to be 2, 3, 5, and 7, respectively. Detail of z-score of each biomarker is shown in Table 1.

### Evaluation of Subtypes and Stages From Subtype Biomarker Progress Sequences

We used SuStaIn as the subtypes-progression model to identify the KOA subtypes, subtype biomarker progress sequences and the probability of assignment to a most probable subtype and stage, respectively, for each knee.

We used 10-fold cross-validation to gain the optimal number of subtypes by the Cross-Validation Information Criterion (CVIC) (16). The CVIC was defined as CVIC = −2 × log *[P (X|M)]*, where *P (X|M)* is the probability of the data *X* for a particular subtypes-progression model *M*.

We tested the differences between the subtypes for a specific biomarker with R (version 3.6.3). The logistic regression was used for binary measurements and a general linear model for continuous or ordered categorical measurements. Then we used *post-hoc* analysis with a SNK test adjustment to test for which subtypes the measurements were different.

We measured the strength of assignment to one of the subtypes in KOA subtypes progression. A strong assignment was defined as that the maximum probability of assigning to a particular subtype is 1.5 times greater than any other two subtypes.

We surveyed the consistency of KL grade and stages from KOA subtypes progression model. KL grade represented the temporal state of knees with KOA roughly. We grouped the knees by KL grade and estimated the probability of assignment to a stage.

### Comparisons Among Subtypes-Progression Model, Subtypes-Only Model, and Stages-Only Model

#### Subtypes-Only Model and Stages-Only Model

We used SuStaIn as the subtypes-progression model to identify the KOA subtypes, subtype biomarker progress sequences, the probability of assignment to a most probable subtype, and stage, respectively, for each knee. The subtypes-only model and stages-only model can also assign knees to a subtype or stage, respectively. The subtype or stage that a knee is assigned reflects the phenotypic or temporal information from our models.

We formulated subtypes-only and stages-only models as close as possible to the subtypes-progression model. Thus, subtypes and stages from the three models (subtypes-only model and stages-only model, subtypes-progression model) are comparable.

In this study, the subtypes-only model was fitted to OA (KL grade ≥2) with a mixture of Gaussians model. The stages-only model was formulated by SuStaIn model, setting the subtype number to 1.

#### Comparisons Among Subtypes and Stages From Three Models

To compare the subtypes and stages from the three models, we put forward a task that separates the knees with/without doubtful KOA (KL grade 0/1) from those with KOA (KL grade ≥2). This task can test the ability of the stages from the three models that separates the knees in time-perspective. The more benefit the stages contribute to the task, the more temporal information are included in the stages. However, if the subtypes have more contributions to the task, it has negative meaning. It indicates that those subtypes include temporal heterogeneity and tend to cluster the knees with similar KOA severity and don't represent true KOA subtypes.

We used logistic regression to compare the subtypes and stages from the three models by the task of separating no or doubtful KOA (KL grade 0/1) from KOA (KL grade ≥2). The input variables were stages and subtypes from the three models and demographic factors: gender, age, BMI and injury. Likelihood ratio comparison between two models was used to assess the goodness of fit of them (17). Statistical significance was set as *P* < 0.05. All were analyzed with R (version 3.6.3).

## Result

There were 678 unilateral knees included, which are from subjects with average age 62.15 years old, 55.01% female, average BMI of 23.1 and 20.65% injury in this study. KL grade 0/1, 2, 3, and 4 accounted for 7.47, 34.22, 40.56, and 17.85%, respectively. 140 knees have injury which was defined as ever injured badly enough to limit ability to walk for at least 2 days.

### KOA Subtypes and Subtype Biomarker Progress Sequences

**Figure 2:**
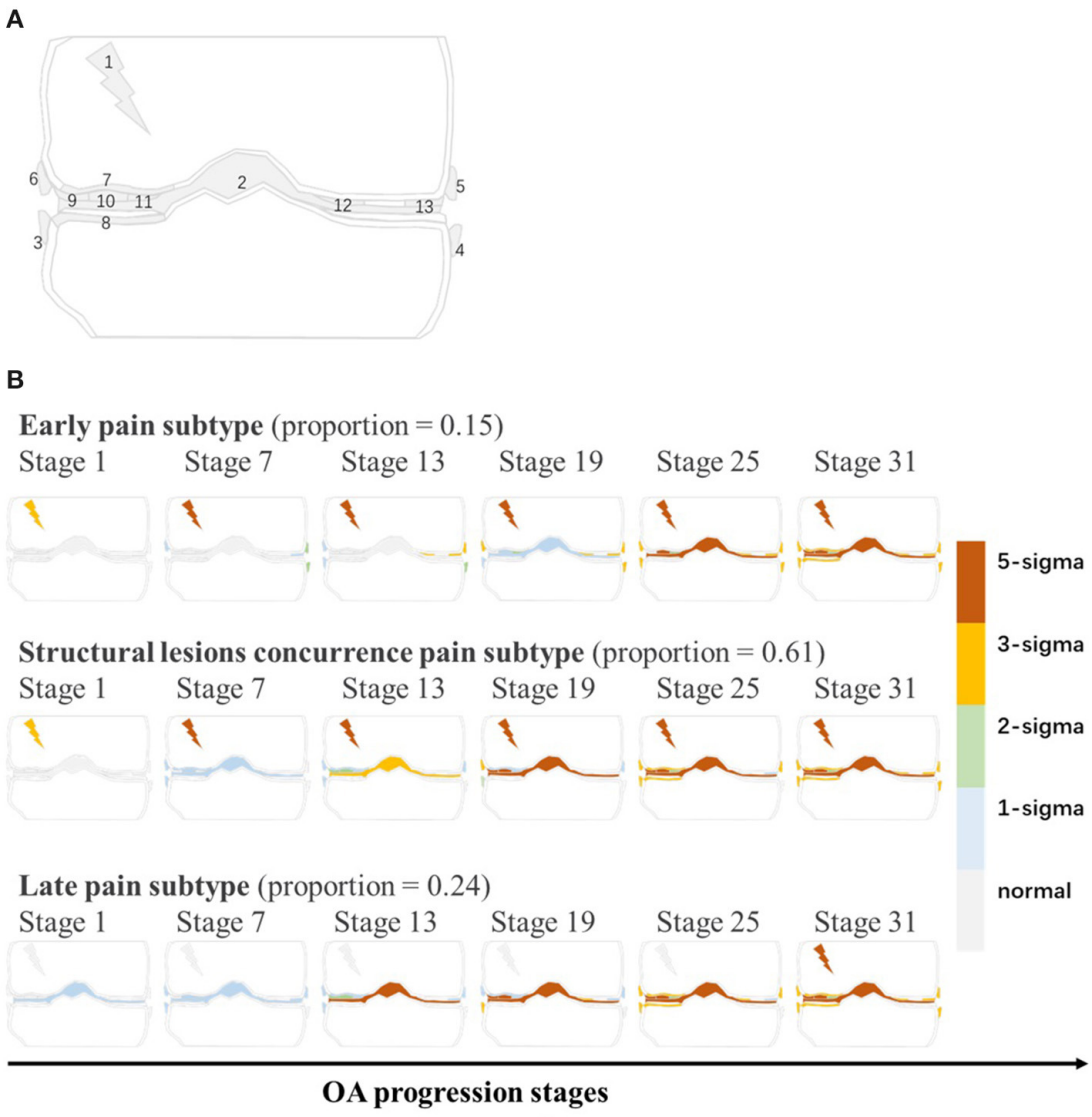
**(A)** The graphical representation of all biomarkers. Gray line is coronal sketch map of knee joint. Numbers in this figure match the order numbers of biomarkers in Table 1. **(B)** KOA subtypes and subtype biomarker progress sequences identified by subtypes-progression model. Proportion of knees assigned to each subtype are shown. Each row represents an biomarkers changes order as KOA stages advance. Biomarkers from different stages reach different z-scores relative to control group. At each stage color in each region indicates level of severity of pain or lesions: gray is unaffected; blue is mildly affected (z-score of 1), and so on.

As is shown in Figure 2, the subtypes-progression model identified three subtypes. Each subtype had different clinical symptoms and structural lesions at different progression stages. We termed three subtypes as early pain, structural lesions concurrence pain and late pain, which account for 15, 61, and 24% of the knees. Early pain subtype could be described as a mild subtype. Serious pain occurs at first stages and osteophytes follows. Not until the latter half stages, other structural lesions occur. In structural lesions concurrence pain, serious pain also occurs at first stages. but all the structural lesions appear and progress soon and almost distribute in almost all the rest stages. Late pain subtype is very similar to the former, but the pain hides till the last stages and structural lesions occur at very early stages.

### Evaluation of Subtypes and Stages From Subtype Biomarker Progress Sequences

#### Demographic and Risk Factors Differences Between Subtypes

All subtypes contained knees of the entire range of the KL grade, except for the early pain subtype on KL grade 0/1 (Table 2). Table 2 shows that early pain subtype had the highest proportion of female (65%) than other two subtypes. BMI in late pain subtype was significantly lowest. Age, injury proportion and gender proportion had no significant difference between the three subtypes.

**Table 2:** Baseline demographics and risk factors for three subtypes and differences between the measurements of all biomarkers used for subtype definitions.

	**Stages *P*-value**	**Subtypes *P*-value**	**Subtype** ^**^**a**^**^		
			**Early pain (*n* = 78)**	**Structural lesions concurrence pain** **(*n* = 419)**	**Late pain (*n* = 181)**
**KL grade**, ***n*****(%)**					
0/1			0 (0)^S, L^	36 (8.59)^E, L^	33 (18.23)^E, S^
2			17 (21.80)^S, L^	149 (35.56)^E^	79 (43.65)^E^
3			40 (51.28)^L^	175 (42.72)^E, L^	58 (32.04)^E^
4			21 (26.92)^E, S^	59 (14.08)^E, L^	11 (6.08)^E, S^
**Injury, yes**, ***n*****(%)**	0.824	0.633	14 (17.95)	88 (21.00)	38 (20.99)
**Gender, female**, ***n*****(%)**	0.294	0.419	50 (64.10)	220 (52.51)	103 (56.91)
**Age, years, mean (standard deviation)**	<0.001	0.023	62.46 (8.67)	61.75 (9.20)	62.96 (9.54)
**BMI, kg/M** ^**2**^ **, mean (standard deviation)**	0.393	−0.004	29.36 (4.79)^L^	29.90 (4.79)^L^	28.02 (4.13)^E, S^
**WOMAC pain score**	<0.001	<0.001	4.96 (3.52)^L^	4.42 (3.27)^L^	0.02 (0.30)^E, S^
**Medial minimum JSW**	<0.001	<0.001	5.53 (1.24)^S, L^	3.1 (1.59)^E, L^	3.9 (1.30)^E, S^
**Osteophytes**					
Tibia medial	<0.001	<0.001	0.73 (0.77)^S, L^	1.21 (0.86)^E^	0.85 (0.71)^E^
Tibia lateral	<0.001	<0.001	2.17 (0.90)^S, L^	0.62 (0.79)^E^	0.49 (0.78)^E^
Femur medial	<0.001	0.778	1.49 (1.29)^S, L^	1.26 (1.13)^E, L^	0.77 (1.02)^E, S^
Femur lateral	<0.001	<0.001	2.40 (0.76)^S, L^	0.69 (0.90)^E^	0.59 (0.93)^E^
**Sclerosis**					
Femur medial	<0.001	<0.001	0.10 (0.38)^S, L^	1.12 (0.94)^E, L^	0.57 (0.76)^E, S^
Tibia medial	<0.001	<0.001	0.15 (0.40)^S, L^	1.12 (0.95)^E, L^	0.53 (0.80)^E, S^
**Mean cartilage thickness**					
Central medial femur (external)	<0.001	<0.001	1.60 (0.42)^S, L^	1.08 (0.49)^E, L^	1.25 (0.35)^E, S^
Central medial femur (center)	<0.001	<0.001	2.39 (0.55)^S, L^	1.62 (0.75)^E, L^	1.96 (0.54)^E, S^
Central medial femur (internal)	<0.001	<0.001	2.10 (0.50)^S, L^	1.80 (0.47)^E^	1.93 (0.38)^E^
Central lateral femur (internal)	<0.001	0.0522	1.70 (0.52)^S, L^	1.86 (0.35)^E^	1.80 (0.36)^E^
Central lateral femur (external)	0.002	<0.001	1.09 (0.59)^S, L^	1.65 (0.35)^E^	1.53 (0.38)^E^

a *Indices is the index number of the subtypes. The subtypes with corner mark ^a^ indicate that the specific subtype is significantly different from the subtypes represented by index numbers (P < 0.05). E, early pain; S, structural lesions concurrence pain; L, late pain*.

After correction for stages, all biomarkers except osteophytes femur medial compartment showed significant differences between the subtypes. *Post-hoc* analysis showed that early pain subtype was mainly different with others. Early pain subtype had more changes in WOMAC pain score. What's more, structural lesions between the lateral and medial compartment were different. More severe lesions occurred in osteophytes from tibia lateral compartment and mean cartilage thickness in lateral femur compartment. Less changes occurred in medial minimum JSW, sclerosis and mean cartilage thickness in central medial subregion. Further, the characteristics between the other subtypes were different. Late pain subtype had slighter change in most structural lesions (WOMAC pain score, medial minimum JSW, osteophytes, sclerosis, and in mean cartilage thickness of central lateral femur subregion).

#### Strength of Assignment to a Subtype

**Figure 3:**
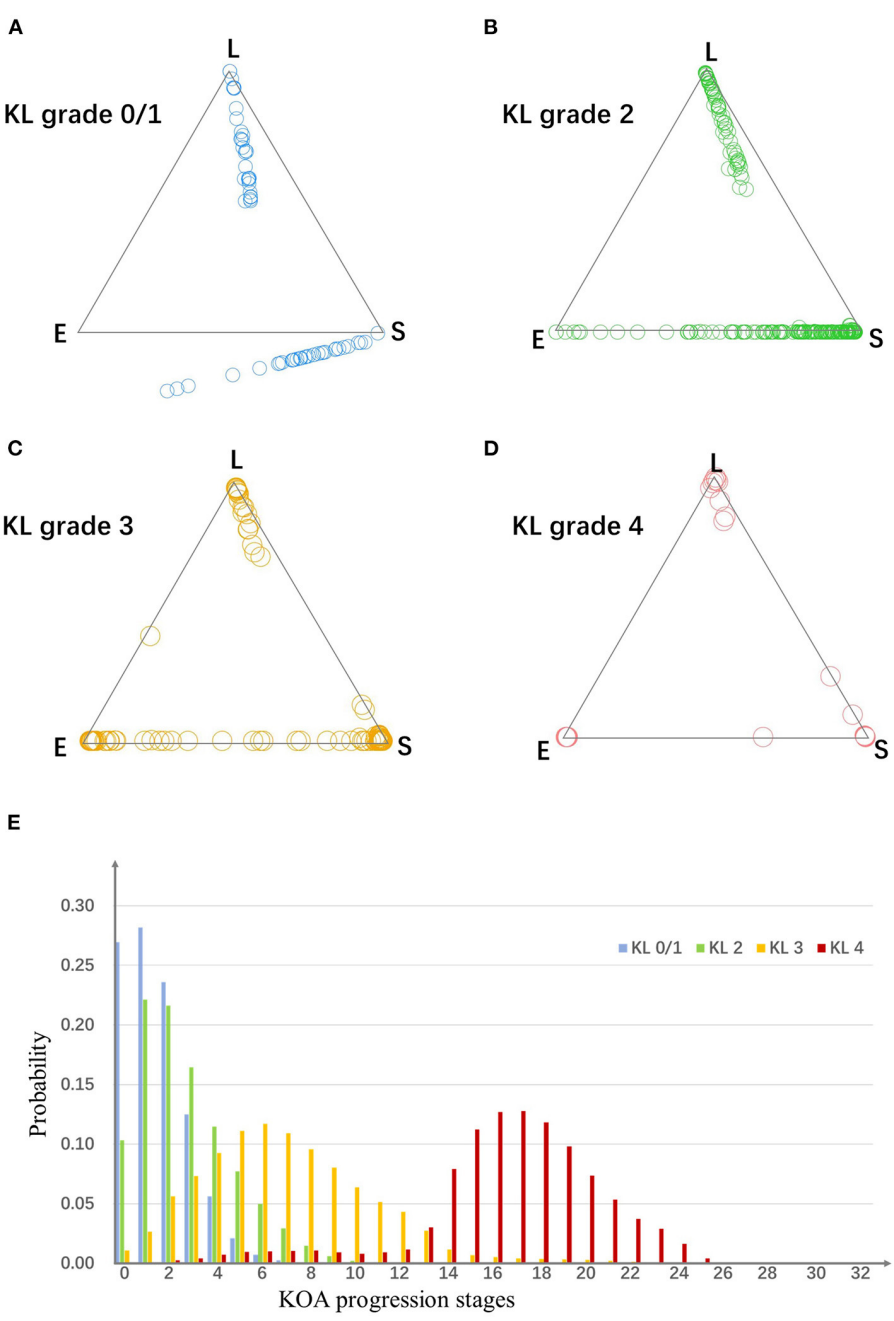
**(A–D)** The assign ability of subtypes and stages. Plots in four scatter plots indicate knees from four KL grade groups. For a triangle scatter plot, each corner indicates a probability of 1 of assigning to a particular subtype, and 0 for the other two subtypes; the center point of the triangle indicates a probability of 1/3 of assigning to each subtype. **(E)** The probability of knees from each KL grade group belong to each stage. E, early pain; S, structural lesions concurrence pain; L, late pain.

As is shown in Figure 3, for OA knees with KL grade 0/1, 2, 3, and 4, the strong assignment to subtypes were 26, 53, 89, and 95%. With disease progress, the strength of assignment in KOA subtypes increased. Moreover, even at early disease stages (KL grade 0/1), 26% of the knees were strongly assigned to a subtype.

#### Probability of Assignment to a Stage

To evaluate the assignment to a particular stage, we estimated the probability knees from each KL grade belonging to each of the stages. As is shown in Figure 3, the knees from different KL grade had different distributions in the stages.

### Comparisons With the Stages-Only Model and Subtypes-Only Model

We used logistic regression model to separate knees with/without doubtful KOA (KL grade 0/1) from those with KOA (KL grade ≥2). Table 3 shows that stages (*p* = 1.08 × 10^−8^) and subtypes (*p* = 0.332) from subtypes-progression model had significant hazards.

**Table 3:** Results for comparing the subtypes and stages from subtypes-progression model with those from subtypes-only and stages-only with logistic regression model by separating knees with no or doubtful KOA (KL grade 0/1) from those with KOA (KL grade ≥2).

	**Subtypes-progression model**		**Stages-only model**		**Subtypes-only model**	
	**β (95% CI)**	**Adjusted *P*-value**	**β (95% CI)**	**Adjusted *P*-value**	**β (95% CI)**	**Adjusted *P*-value**
Intercept	2.82 (−6.26, 0.56)	0.0974	−2.79 (−5.48, −0.14)	0.040	−0.89 (−3.75, 2.13)	0.544
Stages	0.70 (0.49, 0.98)	<0.001	0.32 (0.22, 0.43)	<0.001	—	—
Subtypes	0.29 (−0.29, 0.89)	0.314	—	—	−2.00 (−3.82, −0.81)	0.006
Injury	0.33 (−0.39, 1.12)	0.400	0.43 (−0.25, 1.20)	0.247	0.48 (−0.12, 1.23)	0.181
Gender	0.40 (−0.98, 0.17)	0.158	−0.04 (−0.58, 0.49)	0.872	−0.10 (−0.62, 0.42)	0.729
Age	0.01 (−0.02, 0.04)	0.476	0.03 (0.00, 0.06)	0.029	0.04 (0.01, 0.06)	0.141
BMI	0.05 (−0.01, 0.13)	0.086	0.05 (−0.01, 0.12)	0.089	0.10 (0.04, 0.16)	<0.001

*CI, confidence intervals*.

We compared subtypes-progression model, subtypes-only and stages-only models with likelihood ratio tests. Subtypes-progression model was significantly better fit than subtypes-only (likelihood ratio = 105.59, *p* = 2.2 × 10^−16^) and stages-only (likelihood ratio = 58.04, *p* = 2.57 × 10^−14^) models. A likelihood ratio of above 1 shows that, for distinguish knees with no or doubtful KOA (KL grade 0/1) from those with OA (KL grade ≥2), the subtypes and stages of KOA subtypes progression model provided a significantly better fit than a subtypes-only or stages-only model.

Table 3 shows that the stages in subtypes-progression model had greater β than stages-only model. Rather than subtypes-only model, the subtypes from subtypes-progression model had no statistical significance.

## Discussion

Some studies tried to describe the temporal or phenotypic heterogeneity for KOA. However, those studies that only explain the temporal progression based on the assumption that all the knees came from a single disease progress sequence (1, 4). As KOA is a disease with complex clinical characteristics, this assumption may not hold. Some studies only discussed about phenotypic heterogeneity and don't account for temporal heterogeneity (5–8). Subtypes identified by those studies tend to cluster the knees with similar OA severity, which don't represent true OA subtypes. In our study, we used SuStaIn as a subtypes-progression model to study the temporal or phenotypic heterogeneity simultaneously and constructed a reliable picture of how the lesions spread from a distinct region over the rest of the knee in each subtype.

Because of the limitation of available number of knees, we only discuss a single condition that the unilateral knees have greater KL grade than the contralateral knees at both baseline and all the follow-up 48 months. We fitted the SuStaIn model to 678 knees to identify KOA subtypes and subtype biomarker progress sequences. The biomarkers included the WOMAC pain score, medial minimum JSW, quantitative MRI measures of cartilage thickness, and semi-quantitative radiographic readings (osteophytes and sclerosis, per compartment for the tibia and femur). The subtypes-progression model identified three subtypes and each subtype had its distinct biomarker progress sequence. Three subtypes had different characteristics and were termed as early pain, structural lesions concurrence pain and late pain. The severity of all biomarker increased with greater stages.

Our results are agreed with previous studies, such as bigger pain sore is always associated with severer osteophytes (18–23), and narrower medial minimum JSW is always associated with thinner mean cartilage thickness (24–28).

Between lateral and medial subregion of each subtype, the changes in mean cartilage thickness existed inconsistency. Early pain subtype had significantly slightest changes in medial subregion and significantly greatest changes in lateral subregion. Structural lesions concurrence pain subtype had opposite changes. Late pain subtype had medium changes in both subregion. Some studies found that greater BMI is associated with incident medial tibiofemoral OA (3) and more serious changes in lateral subregion in mean cartilage thickness, which is consistent with our results.

The existence of phenotypic heterogeneity of KOA is proved by our study. When we measured the strength of a knee's assignment to a given subtype, it showed strong assignment of KOA patients to the subtypes. Therefore, explaining heterogeneity in this study about KOA progression is very necessary.

The subtypes and stages we identified have power to separate the knees with phenotypic or temporal heterogeneity. At no or doubtful KOA (KL grade 0/1), many knees gather around the vertices of the triangles. It shows the subtypes are so effective that have the ability of identifying knees even in very early stages. Besides, the stages are certified to have the power to separate the knees with different disease severity. The probabilities of knees with each KL grade belonging to each of the stages show that the distribution of the stages differ between KL grades.

The subtypes and stages from subtypes-progression model performed significantly better than subtypes-only and stages-only models. We used logistic regression to compare the subtypes and stages from the three models by the task of separating no or doubtful KOA (KL grade 0/1) from KOA (KL grade ≥2). With the temporal task of separating early disease stages (KL grade 0/1) from OA (KL grade ≥2), it shows that subtypes and stages from subtypes-progression model are close to true KOA subtypes and stages. Bigger regression coefficient of stages from subtypes-progression model shows that they contain more complete temporal information. Furthermore, rather than those from subtypes-only model, subtypes from subtypes-progression model have no contribution to this temporal task. It suggests that the subtypes from subtypes-progression model contain hardly any temporal heterogeneity and are close to true OA subtypes.

The subtypes and stages identified by this study have clinical practice value. First, with our subtypes-progression model, each knee can be assigned into certain subtype, suggesting particular disease characteristics. So that, we can help doctor to identify the significant features of each knee and make more proper treatment plan. Second, whole KOA progress course is divided into more detailed stages. They offer a mini scale for doctor to learn disease progress statue of a knee. What's more, the biomarker progress sequence can show the progress pathway for each knee. The doctors and patients can foresee their subsequent biomarker changes and estimate if a knee maintain same stage over a period of time. It has a significant meaning in chronic disease management. And finally, the study method can also be expanded to carry out the research of other chronic disease.

There are some limitations in this study. As being confined to the quantity of the knees available, the study population only included the knee that afflicted with KOA earlier and had greater KL grade for every knee. So our results may only act as a reference for the knee with more serious OA condition of the subject. In addition, the biomarkers only contain the pain score and image assessment data. In future work, we can study other groups of knees that afflicted with KOA later and remain smaller KL grade or alternately having smaller KL grade, and try to analyze more categories of biomarkers, e.g., biochemical biomarker measurements from serum and urine samples, so that we can learn the KOA progression in wider range.

## Conclusion

The subtypes-progression model identifies three subtypes and each subtype has its distinct biomarker progress sequence. There exists phenotypic heterogeneity of KOA, bigger pain sore is always associated with severer osteophytes and the changes in mean cartilage thickness exist inconsistency between lateral and medial subregions of each subtype. The subtypes and stages from subtypes-progression model have power to separate the knees with phenotypic or temporal heterogeneity and perform significantly better than those from stages-only model and subtypes-only model. In a word, with subtypes-progression model, stages contain more complete temporal information and subtypes are close to real OA subtypes.

## Data Availability Statement

Publicly available datasets were analyzed in this study. This data can be found here: https://nda.nih.gov/oai/.

## Ethics Statement

The original OAI participant recruitment and data collection process have obtained ethical approval and informed consent. No specific ethical approval was required for this study.

## Author Contributions

ML participated in the design of the study, analysis and interpretation of the data, and drafting of the article. LL participated in analysis of data, manuscript preparation, and critical revision of the article for important intellectual content. JL participated in the design of the study and analysis of the data. LP participated in analysis and interpretation of the data. XZ participated in design of the study and critical revision of the article for important content. XL participated in interpretation of the data and drafting of the article. All authors read and approved the final manuscript.

## Conflict of Interest

The authors declare that this study received funding from the Center of Excellence-International Collaboration Initiative Grant (No. 139170052) and the 1.3.5 Project for Disciplines of Excellence, West China Hospital, Sichuan University (No. ZYJC18010). The funder was not involved in the study design, collection, analysis, interpretation of data, the writing of this article or the decision to submit it for publication.

## Publisher's Note

All claims expressed in this article are solely those of the authors and do not necessarily represent those of their affiliated organizations, or those of the publisher, the editors and the reviewers. Any product that may be evaluated in this article, or claim that may be made by its manufacturer, is not guaranteed or endorsed by the publisher.
